# Psychopharmacological Treatments for Mental Disorders in Patients with Neuromuscular Diseases: A Scoping Review

**DOI:** 10.3390/brainsci12020176

**Published:** 2022-01-28

**Authors:** Chiara Brusa, Giulio Gadaleta, Rossella D’Alessandro, Guido Urbano, Martina Vacchetti, Chiara Davico, Benedetto Vitiello, Federica S. Ricci, Tiziana E. Mongini

**Affiliations:** 1 Section of Child and Adolescent Neuropsychiatry, Department of Public Health and Pediatric Sciences, University of Turin, 10126 Turin, Italy; chiara.brusa@unito.it (C.B.); rossella.dalessandro@unito.it (R.D.); mvacchetti@cittadellasalute.to.it (M.V.); chiara.davico@unito.it (C.D.); benedetto.vitiello@unito.it (B.V.); federica.ricci@unito.it (F.S.R.); 2 Neuromuscular Unit, Department of Neurosciences “Rita Levi Montalcini”, University of Turin, 10126 Turin, Italy; giulio.gadaleta@unito.it (G.G.); guido.urbano@edu.unito.it (G.U.)

**Keywords:** neuromuscular diseases, mental disorders, psychiatric symptoms, psychopharmacological treatments

## Abstract

Mental disorders are observed in neuromuscular diseases, especially now that patients are living longer. Psychiatric symptoms may be severe and psychopharmacological treatments may be required. However, very little is known about pharmacotherapy in these conditions. We aimed to summarize the current knowledge on the use of psychopharmacological treatments for mental disorders in patients living with a neuromuscular disease. A scoping review was performed using the methodology of the Joanna Briggs Institute. Four databases were searched from January 2000 to July 2021. Articles were screened based on titles and abstracts. Full-text papers published in peer-reviewed journals in English were selected. Twenty-six articles met eligibility criteria, all being case reports/series focusing on the psychopharmacological control of psychiatric symptoms for the following conditions: myasthenia gravis (*n* = 11), Duchenne (*n* = 5) and Becker (*n* = 3) muscular dystrophy, mitochondrial disorders (*n* = 3), glycogen storage disease (*n* = 1), myotonic dystrophy (*n* = 1), hyperkalemic periodic paralysis (*n* = 1), and congenital myasthenic syndrome (*n* = 1). None of the articles provided details on the decision-making process to choose a specific drug/regimen or on follow-up strategies to monitor safety and efficacy. Larger studies showing real-world data would be required to guide consensus-based recommendations, thus improving current standards of care and, ultimately, the quality of life of patients and their families.

## 1. Introduction

Neuromuscular diseases encompass a wide range of disorders due to genetic or acquired etiologies and are characterized by the anatomic localization of the pathology within the motor unit. The motor unit consists of the motor neuron in the ventral horns of the spinal cord and brainstem motor nuclei, the peripheral nerve, the neuromuscular junction, and the muscle fiber. Defects in any of these components result in weakness, a common feature of motor neuron disorders, neuropathies, neuromuscular junction disorders, and muscle fiber disorders (the latter being further subdivided into categories based on histopathology features on muscle biopsy: dystrophies, congenital myopathies, mitochondrial diseases, metabolic disorders affecting the muscle, inflammatory myopathies, and infectious myositis).

Each neuromuscular disease is “rare” according to the European Union definition of a disease that affects no more than 1 person in 2000, and some of them are very rare. Yet, collectively, these conditions are quite common [1], and a general pediatrician/practitioner will care for at least one neuromuscular patient on average at any one time. In addition, with more patients being now defined genetically [2], the incidence and prevalence rates for some disorders have increased from earlier pregenetic studies. On top of that, prevalence rates directly reflect duration of life, and the introduction and adoption of supportive care options for neuromuscular disorders over the last decades [3,4,5,6,7] have contributed to improving the overall survival of these patients. Furthermore, the recent availability of innovative drug therapies for some patients—in particular those with infantile-onset Pompe disease (IOPD) [8] and spinal muscular atrophy (SMA) type 1 [9,10,11]—have further contributed to prolonging their survival.

Overall, neuromuscular diseases may present from prenatal development to adulthood, and are usually characterized by a chronic course—especially when the underlying origin is a genetic defect. Physical impairment and morbidity are often substantial, and motor difficulties may be complicated by respiratory, cardiac, nutritional, and skeletal compromise, with resultant impaired independent functioning throughout life. Therefore, combined efforts of multiple specialists are often required to evaluate and manage such patients to optimize their potential and quality of life.

Now that patients with neuromuscular diseases are living longer, more and more attention has been paid to their health-related quality of life and psychological care. People living with chronic medical conditions—as most people with a neuromuscular disease—are at higher risk of developing mental disorders like anxiety and depression. To investigate the effect of the burden of the disease on mental health, patient reported outcome measures (PROM) and quality of life (QOL) instruments are available, both general and disease-specific ones, e.g., for Spinal Muscular Atrophy (SMA) and Duchenne Muscular Dystrophy (DMD) [12,13,14,15]. For the pediatric population, questionnaires for parents are available to report information about their child, and age-specific questionnaires using appropriate language for that child’s development have been created and validated [16,17].

In addition to the mental disorders generally experienced by people living with a chronic disease, specific comorbid neurodevelopmental or other psychiatric disorders have been described as part of the clinical phenotype of some neuromuscular diseases. High rates of intellectual disability—ID (17–27%), learning disabilities (26%), autism spectrum disorder—ASD (15%), attention-deficit hyperactivity disorder—ADHD (32%), and anxiety (27%) have been reported in people with DMD [5,18,19,20]. Similarly, ASD, ADHD, alexithymia, and other behavioral problems have been described in patients with Myotonic Dystrophy type 1 (DM1) [21,22]. Published data for over nearly half of a century have demonstrated an association between Myasthenia Gravis (MG) and mood disorders [23]. Patients with mitochondrial disorders can present with primary psychiatric symptomatology, including anxiety, depression, bipolar disorder, psychosis, and obsessive-compulsive disorder, which are mainly described in those with a diagnosis of mitochondrial encephalomyopathy with lactic acidosis and stroke-like episodes (MELAS) [24].

Patients with psychiatric or behavioral issues should be referred to a mental health care professional for assessment and management. Milder symptoms may benefit from nonpharmacological interventions. Should these interventions not be effective, e.g., due to the increased severity of symptoms, psychopharmacological treatments might be required. However, very little is known about psychopharmacological management of mental disorders in patients with neuromuscular diseases, as regards to both their efficacy and safety profile. Studies on real world data in large populations are lacking. No guidelines for clinicians dealing with neuromuscular patients are available in terms of recommended medications (type, regimen, drug interactions) and follow-up schedules to monitor clinical outcomes and adverse events. Such guidelines would be of significant help for neuromuscular patients who might experience more frequent and/or more severe adverse events due to their comorbidities, especially from a cardiac point of view, and due to the interferences with the considerable number of medications they might already be on.

In this scoping review, we first aim to explore and summarize the current knowledge on psychopharmacological treatments for mental disorders in patients with neuromuscular diseases. Secondly, by providing up-to-date information on the use of psychiatric medications in real-world neuromuscular settings and on available recommendations/expert opinions, we aim to raise awareness on this topic. We believe sharing data on day-to-day management of patients living with a neuromuscular conditions and experiencing a mental disorder would add significant insight into ways to improve the current standards of care for neuromuscular diseases and, in turn, the quality of life of patients and their families.

## 2. Materials and Methods

The Joanna Briggs Institute (JBI) methodology for scoping reviews, described in the online JBI Reviewer’s Manual [25], was employed to conduct the review. The results are presented following the Preferred Reporting Items for Systematic reviews and Meta-Analyses extension for Scoping Reviews (PRISMA-ScR) Checklist [26]. No a priori protocol was registered. Further information on the process can be obtained from the corresponding author on request.

### 2.1. Inclusion Criteria

The inclusion criteria used to select the articles for this review are based on the Population, Concept, and Context (PCC) elements reported below.

Population. We searched for articles reporting the use of medications for mental disorders in patients with neuromuscular conditions including spinal muscular atrophies, neuromuscular junction disorders (myasthenia gravis, congenital myasthenic syndromes), muscular dystrophies (dystrophinopathies, congenital muscular dystrophies, limb-girdle muscular dystrophies, facioscapulohumeral dystrophy, myotonic dystrophies), structural congenital myopathies, non-dystrophic myotonias, mitochondrial myopathies, glycogen storage diseases, and myositis. For this review, we did not consider neuropathies, fibromyalgia, or motor neuron disorders other than spinal muscular atrophies. As regards to the age range, we included articles considering both the adult and the pediatric population.

Concept. We selected articles analyzing the use of the following psychopharmacological agents: anxiolytics, antidepressants, antipsychotics, and central nervous system stimulants.

Context. No cultural, geographical, race, or gender-specific limits were considered for our review, with the reason being that there are no previous data suggesting any differences in the pharmacological effects of these agents according to the above mentioned categories.

### 2.2. Search Strategy

The review covers data published between January 2000 and July 2021. Selected keywords were combined to create search strategies, adjusted for each screened database. Articles were searched in the following databases: PubMed/MEDLINE, Embase, PsycINFO, and Scopus. Search terms included: “neuromuscular diseases”, “spinal muscular atrophies”, “neuromuscular junction disorders”, “myasthenia gravis”, “congenital myasthenic syndromes”, “muscular dystrophies”, “limb-girdle muscular dystrophies”, “facioscapulohumeral dystrophy”, “structural congenital myopathies”, “myotonic dystrophies”, “non-dystrophic myotonias”, “myositis”, “inflammatory myopathies”, “mitochondrial myopathies”, “glycogen storage diseases”, “psychopharmacology”, “psychotropic drugs”, “anti-anxiety agents”, “anti-depressive agents”, “antipsychotic agents”, “central nervous system stimulants”, “mental disorders”, “anxiety disorders”, “obsessive-compulsive disorder”, “panic disorder”, “bipolar disorder”, “mood disorders”, “depressive disorder”, “neurodevelopmental disorders”, “attention deficit disorder with hyperactivity”, “conduct disorder”, “autism spectrum disorder”, “psychotic disorders”, and “schizophrenia” Supplementary Material File S1 (online supporting information) shows the search process (search string and search terms) used to retrieve the final articles from PubMed/MEDLINE. References from relevant articles were searched for inclusion of additional papers not previously identified through the systematic search.

### 2.3. Screening and Selection of Articles

Articles were initially screened based on titles and abstracts according to the PCC elements previously described. Duplicates were removed. Only full-text papers published after 2000 in peer-reviewed journals and in the English language were selected. The articles were examined by two authors (CB and GG), and eligibility for inclusion was performed independently; in case of discordant opinion between the reviewers, the eligibility of the article was discussed until consensus was reached.

### 2.4. Extraction and Presentation of Results

All data relevant to inform the scoping review objectives and questions were extracted and are summarized in Table 1, Table 2, Table 3, Table 4, Table 5, Table 6, Table 7 and Table 8. The strength of evidence for each article was assessed according to the Levels of Evidence developed by the Joanna Briggs Institute (JBI) [25]. Results were grouped according to the following neuromuscular diseases: myasthenia gravis, Duchenne muscular dystrophy, Becker muscular dystrophy, MELAS, glycogen storage disease type 1, myotonic dystrophy type 2, hyperkalemic periodic paralysis, and congenital myasthenic syndrome (Table 1, Table 2, Table 3, Table 4, Table 5, Table 6, Table 7 and Table 8).

## 3. Results

A total of 26 articles on the use of psychiatric medications for mental disorders in patients with a neuromuscular disease were included after screening and selection, focusing on the control of various behavioral and emotional symptoms in the context of myasthenia gravis (*n* = 11), Duchenne muscular dystrophy (*n* = 5), Becker muscular dystrophy (*n* = 3), MELAS (*n* = 3), glycogen storage disease type 1 (*n* = 1), myotonic dystrophy type 2 (*n* = 1), hyperkalemic periodic paralysis (*n* = 1), and congenital myasthenic syndrome (*n* = 1).

### 3.1. Myasthenia Gravis (MG)

We found 11 articles reporting on the use of psychopharmacological treatments in patients suffering from anxiety, somatic, depressive, psychotic, or bipolar disorder symptoms, or a combination of two or more psychiatric symptoms. In particular, we found 10 case reports and a case series, as shown in Table 1.

### 3.2. Duchenne Muscular Dystrophy (DMD)

Five articles reported on psychopharmacological treatments of neurodevelopmental disorders (i.e., ID, ASD, ADHD, tic disorders) often aimed at controlling associated behavioral and emotional symptoms. Two papers were case reports, 2 were case series, and 1 was an observational study with no control group, as shown in Table 2.

### 3.3. Becker Muscular Dystrophy (BMD)

Three articles reported on medications to treat symptoms of neurodevelopmental, anxiety, psychotic, and depressive disorders in 2 case reports and a case series, as shown in Table 3.

### 3.4. Mitochondrial Encephalomyopathy with Lactic Acidosis and Stroke-like Episodes (MELAS)

We found 2 case reports and 1 case series on the use of psychiatric medications for depressive, psychotic, and obsessive-compulsive symptoms, respectively, as shown in Table 4.

### 3.5. Glycogen Storage Disease Type 1 (GSD-1)

One case report documented the use of psychiatric medications to control psychotic symptoms in a patient with GSD-1, as shown in Table 5.

### 3.6. Myotonic Dystrophy Type 2 (DM2)

We found 1 case report detailing the use of psychopharmacological treatments to control psychotic symptoms in a patient with DM2, as shown in Table 6. 

### 3.7. Hyperkaliemic Periodic Paralysis (HPP)

One case report documented the use of multiple psychopharmacological treatments for bipolar disorder in a patient with HPP, as shown in Table 7.

### 3.8. Congenital Myasthenic Syndrome (CMS)

We found 1 case report on the use of antidepressants for major depressive symptoms in a patient with a CMS, as shown in Table 8.

## 4. Discussion

This review confirms that data published over the last 20 years on the use of psychopharmacological treatments for mental disorders in patients with neuromuscular diseases are scarce. Only 26 articles have been found on this topic, generally with low level of evidence as most of them are case reports or very small case series/observational studies, with the exception of 2 large case series. However, even these two larger studies did not detail the decision making process leading to the choice of a specific psychiatric drug or regimen, nor did they describe follow-up strategies to monitor efficacy and safety.

The scarcity of data is in contrast with the high frequency of psychiatric comorbidities reported for many neuromuscular diseases. As already mentioned above, chronic medical conditions—such as neuromuscular disorders—represent significant risk factors for developing depression and anxiety. In literature, symptoms of depression and anxiety have been reported among people living with a neuromuscular disorder, especially those with MG [23,53]. In addition to depression and anxiety, neurodevelopmental disorders such as ASD and ADHD—with or without comorbid intellectual disability and other behavioral and emotional difficulties—have been reported in DMD, BMD [39,43], and DM1 [54]. Psychiatric symptoms may be a manifestation of primary mitochondrial disorders such as MELAS [55]. It is worth considering that mental health disorders due to living with a disability—e.g., depression and anxiety—and psychiatric symptoms, which are part of the neuromuscular phenotype, are different entities likely requiring different approaches. Similarly, mental disorders due to living with a life-limiting disease like DMD might deserve different treatment strategies as compared to the ones observed in neuromuscular diseases with normal life expectancy (e.g., MG).

Currently, specific recommendations for clinicians are in place only for conditions such as DMD [5] and DM1 [21,22]. Mental health and quality of life screening is suggested for these patients at each neuromuscular clinic visit, and the involvement of a mental health clinician is warranted for further assessment and management in the case of a positive screening. However, authors agree on the fact that there is little research, in terms of real-world data and guidelines, that clinicians can rely on when taking care of neuromuscular patients with moderate-to-severe psychiatric manifestations. Management of such symptoms with psychosocial and/or psychotherapeutic approaches alone can be significantly challenging, and a psychopharmacological intervention may be required.

The use of psychiatric drugs in neuromuscular patients may be hampered by multiple factors. As regards to efficacy monitoring, there is no consensus on the standardized outcome measures that could be used to evaluate the effect of psychiatric medication in this population. As to safety monitoring, patients with neuromuscular diseases are already at risk of multisystemic complications, especially considering their cardiac involvement, which, in turn, may represent a contraindication to the administration of psychiatric medications (e.g., some antidepressants, atypical antipsychotics, or central nervous system stimulants). On top of that, the longer the patients survive, the higher the risk of more severe complications and, therefore, the more complex the management of mental disorders. This is particularly true for specific populations such as adults living with DMD, as highlighted during an ENMC International Workshop in 2014 [56], and in the recently published consensus guidelines for adults with DMD produced by the UK Adult North Star Network [57]. In addition, neuromuscular patients may be already on medications that can worsen psychiatric symptoms (e.g., corticosteroids for DMD and MG). Additionally, patients may present neuromuscular conditions for which a number of medications are contraindicated. This is the case, for instance, of primary mitochondrial diseases. On this regard, an international Delphi-based consensus has been published in 2020 [58]. The authors of this document expressed a strong consensus on the safe use of antidepressants, antipsychotics, and benzodiazepines in this population when clinically indicated. However, they also agreed on the lack of large studies, making it impossible to directly draw recommendations for the clinical practice from their consensus.

We believe that this scoping review identifies a significant gap in the knowledge of psychopharmacological treatments for mental disorders in patients with neuromuscular diseases. To date, the limited number of reports and the overall low level of evidence of published data do not allow us to make recommendations on this topic. Large longitudinal studies would be strongly required to share clinical experiences and gather more insights on the management of psychopharmacological medications in patients with neuromuscular diseases. Standardized outcome measures should be used to objectively evaluates outcomes, and longitudinal follow-up should be planned to monitor side effects. Multidisciplinary efforts, especially involving mental health professionals (psychiatrists, psychologists) and cardiologists, are fundamental to minimize adverse events.

Considering that mental health status is strongly related to the quality of life of patients and their families, raising awareness on this topic is becoming of utmost importance now that patients are living longer thanks to the improvements in standards of care and the availability of innovative drug therapies.

## 5. Conclusions

In conclusion, mental disorders are commonly observed in people living with a neuromuscular disorder, in both the pediatric and the adult population. As shown by our review, psychiatric symptoms may be severe, and psychopharmacological treatments may be required. Administering psychotropic drugs might be challenging in neuromuscular patients who are already at risk of developing complications, especially cardiac ones, and who might already be on several other medications. Larger studies showing real-world data would be required to guide consensus-based recommendations, thus improving the current standards of care and, ultimately, the quality of life of patients living with a neuromuscular disease and their families.

## Figures and Tables

**Table 1:** Psychopharmacological treatments use in Myasthenia Gravis (MG).

Authors, Year	Study Design	Sample (Size, Sex, Age)	Psychiatric Disorders/Symptoms	Psychiatric Medications	Symptoms Improvement (Yes/No/Not Reported)	Side Effects	Level of Evidence [25]
Kalita et al., 2020 [27]	Case report	*n* = 1 M 54 years	Anxiety symptoms	^a^ BDZ (alprazolam)	Yes	None	4.d
Jordan and Ortiz, 2019 [28]	Case report	*n* = 1 F 31 years	Anxiety symptoms Depressive symptoms ADHD ^b^ PTSD Polysubstance abuse	^c^ SARIs (trazodone) Imidazopyridine derivative (zolpidem) BDZ (alprazolam) Alpha-blockers (doxazosin then prazosin)	No (trazodone, zolpidem) Yes (alprazolam but abuse) Yes (doxazosin then prazosin)	Alprazolam: symptoms consistent with withdrawal syndrome when drug stopped after 11 years	4.d
Yamamoto et al., 2018 [29]	Case series	*n* = 2 F 69 and 64 years	Patient 1: somatic symptom disorder Patient 2: depressive symptoms	Patient 1: tetracyclic antidepressant (mianserin) Patient 2: ^d^ SSRIs (paroxetine), BDZ (alprazolam, flunitrazepam)	Patient 1: Yes Patient 2: Yes	None	4.c
Kyllo et al., 2017 [30]	Case report	*n* = 1 F In her thirties	Psychotic symptoms (postpartum)	Second generation antipsychotic (quetiapine then olanzapine)	Yes (olanzapine)	Quetiapine: excessive sedation	4.d
She et al., 2017 [31]	Case report	*n* = 1 F 23 years	Psychotic symptoms	Second generation antipsychotic (olanzapine then paliperidone) BDZ	Yes (paliperidone)	Olanzapine: dystonia, dysphagia, breathing difficulties requiring tracheotomy BDZ: respiratory distress	4.d
Al-Hashel et al., 2016 [32]	Case report	*n* = 1 F 29 years	Psychotic symptoms Major depressive disorder	Second generation antipsychotic (paliperidone, then long-acting risperidone, then aripiprazole) ^d^ SSRIs (fluoxetine then escitalopram)	No (paliperidone, risperidone) Not reported (aripiprazole, fluoxetine, escitalopram)	Long-acting risperidone: worsening of MG symptoms with respiratory distress not responding to ^e^ IVIG and requiring ventilation and plasma exchange	4.d
Kim et al., 2013 [33]	Case report	*n* = 1 F 46 years	Psychotic symptoms	Second generation antipsychotic (aripiprazole, quetiapine, paliperidone) First generation antipsychotic (haloperidol) Phenothiazine antipsychotic (chlorpromazine)	No (aripiprazole, quetiapine, paliperidone) Yes (haloperidol but side effects) Yes (chlorpromazine)	Haloperidol: lower extremity tremor; therefore, procyclidine added but respiratory distress	4.d
Wilson and Ferguson, 2013 [34]	Case report	*n* = 1 M 64 years	Bipolar disorder	Second generation antipsychotic (olanzapine) Mood stabilizer (sodium valproate) First generation antipsychotic (zuclopenthixol decanoate)	No (olanzapine) No (sodium valproate) No (zuclopenthixol decanoate)	Sodium valproate: stopped due to marked peripheral oedema Zuclopenthixol decanoate stopped due to uncovering of MG symptoms	4.d
Chiu et al., 2011 [35]	Case report	*n* = 1 F 26 years	Psychotic symptoms	Second generation antipsychotic (quetiapine, then clozapine)	Not reported	Quetiapine: general weakness, hoarseness, dysarthria, dysphagia, dyspnoea Clozapine: dysphonia, dysphagia, ptosis, respiratory failure requiring invasive ventilation	4.d
Alevizos et al., 2006 [36]	Case report	*n* = 1 M 33 years	Bipolar disorder	^d^ SSRIs (citalopram) Lithium Mood stabilizer (sodium valproate)	No (citalopram) Yes (lithium but side effects) Yes (sodium valproate)	Lithium: stopped due to uncovering of MG symptoms (severe generalized weakness) despite improved mood	4.d
Shinkai et al., 2001 [37]	Case report	*n* = 1 M 39 years	Major depressive disorder	^d^ SSRIs (fluvoxamine)	Not reported	Fluvoxamine stopped due to dysphagia and aspiration pneumonia	4.d

^a^ BDZ: benzodiazepine; ^b^ PTSD: post-traumatic stress disorder; ^c^ SARIs: serotonin antagonist and reuptake inhibitors; ^d^ SSRIs: selective serotonin reuptake inhibitor; ^e^ IVIG: Intravenous Immunoglobulin.

**Table 2:** Psychopharmacological treatments use in Duchenne Muscular Dystrophy (DMD).

Authors, Year	Study Design	Sample (Size, Sex, Age)	Psychiatric Disorders/Symptoms	Psychiatric Medications	Symptoms Improvement (Yes/No/Not Reported)	Side Effects	Level of Evidence [25]
Noda et al., 2021 [38]	Case report	*n* = 1 M 17 years	ID ASD Psychotic symptoms	Second generation antipsychotic (aripiprazole)	Yes	None	4.d
Darmahkasih et al., 2020 [39]	Case series	*n* = 700 698 M, 2 F mean age (SD): 13 years (5.6)	ASD ADHD ID Specific learning disabilities Motor and/or vocal tics Obsessive-compulsive symptoms Anxiety symptoms Depressive symptoms Emotional and behavioral dysregulation	^a^ SSRIs (196/700) ^b^ SNRIs (8/700) Other antidepressants (9/700) CNS stimulants (86/700) Non-stimulants (44/700) Antipsychotics (19/700) Other medications: (22/700)	Not reported	Not reported	4.c
Lionarons et al., 2019 [40]	Observational study with no control group	*n* = 10 M 6.3–9.8 years	ADHD	CNS stimulant (methylphenidate)	Yes (7/10) No (3/10)	No major side effects	3.e
Lee et al., 2018 [41]	Case series	*n* = 15 M 5–23 years	Obsessive-compulsive symptoms Anxiety symptoms (11/15)	^a^ SSRIs (fluoxetine or paroxetine or sertraline or citalopram or escitalopram)	Yes (10/15) No (2/15) Not reported (3/15)	1/15: rash, ^c^ GI upset, apathy, urinary urgency with all SSRIs but paroxetine	4.c
Hendriksen et al., 2016 [42]	Case report	*n* = 1 M 9 years	Obsessive-compulsive symptoms ASD ID (borderline ^d^ IQ)	^a^ SSRI (fluoxetine)	Yes	None	4.d

^a^ SSRIs: selective serotonin reuptake inhibitors; ^b^ SNRIs: serotonin and norepinephrine reuptake inhibitors; ^c^ GI: gastrointestinal; ^d^ IQ: intelligence quotient.

**Table 3:** Psychopharmacological treatments use in Becker Muscular Dystrophy (BMD).

Authors, Year	Study Design	Sample (Size, Sex, Age)	Psychiatric Disorders/Symptoms	Psychiatric Medications	Symptoms Improvement (Yes/No/Not Reported)	Side Effects	Level of Evidence [25]
Lambert et al., 2020 [43]	Case series	*n* = 70 M 1.0–36.7 years	ASD ADHD ID Specific learning disabilities Motor and/or vocal tics Obsessive-compulsive symptoms Anxiety symptoms Depressive symptoms Emotional and behavioral dysregulation	^a^ SSRIs (6/70) CNS stimulants (7/70) Non-stimulants (8/70) Antipsychotic aripiprazole (4/70) ^b^ BDZ clonazepam (1/70) Mood stabilizer oxcarbazepine (1/70)	Not reported	Not reported	4.c
Fernandes Santos, 2019 [44]	Case report	*n* = 1 M 50 years	Psychotic symptoms Depressive symptoms	Second generation antipsychotic (aripiprazole) Tetracyclic antidepressant (mirtazapine) ^b^ BDZ (alprazolam)	Yes	None	4.d
Chaichana et al., 2007 [45]	Case report	*n* = 1 M 35 years	Major depressive disorder Cluster B personality traits (histrionic behavior)	^a^ SSRIs (fluoxetine, sertraline, citalopram) Second generation antipsychotic (risperidone, aripiprazole) Tricyclic antidepressant (nortriptyline) ^c^ SARI (trazodone) ^d^ SNRI (duloxetine)	No (fluoxetine, sertraline, citalopram, risperidone, aripiprazole, nortriptyline, trazodone) Yes (duloxetine, trazodone in addition to behavioral therapy)	None	4.d

^a^ SSRIs: selective serotonin reuptake inhibitors; ^b^ BDZ: benzodiazepine; ^c^ SARIs: serotonin antagonist and reuptake inhibitors; ^d^ SNRIs: serotonin and norepinephrine reuptake inhibitors.

**Table 4:** Psychopharmacological treatments use in Mitochondrial Encephalomyopathy with Lactic Acidosis and Stroke-like episodes (MELAS).

Authors, Year	Study Design	Sample (Size, Sex, Age)	Psychiatric Disorders/Symptoms	Psychiatric Medications	Symptoms Improvement (Yes/No/Not Reported)	Side Effects	Level of Evidence [25]
Cozart et al., 2018 [46]	Case report	*n* = 1 F 46 years	Major depressive disorder	^a^ SNRI (duloxetine)	Yes (low dose: 20 mg/day)	Higher dose (40 mg/day): loss of bowel control	4.d
Grover et. al, 2006 [47]	Case report	*n* = 1 M 16 years	Psychotic symptoms	Multiple mood stabilizers (anticonvulsants), antipsychotics, antidepressants by a general practitioner; then: ^b^ BDZ (diazepam) Second generation antipsychotic (quetiapine)	Not reported (multiple anticonvulsants, antipsychotics, antidepressants) Yes (diazepam, quetiapine)	Multiple anticonvulsants, antipsychotics, antidepressants: drooling of saliva, painful hyperextension of neck and back, rigidity, staring, stereotypic hand movements, mutism, impairment in daily life activities	4.d
Lacey and Salzberg, 2008 [48]	Case series	*n* = 2 M 30 and 51 years	Obsessive-compulsive symptoms	^c^ SSRIs Second generation antipsychotic (quetiapine, olanzapine)	No	None	4.c

^a^ SNRIs: serotonin and norepinephrine reuptake inhibitors; ^b^ BDZ: benzodiazepine; ^c^ SSRIs: selective serotonin reuptake inhibitors.

**Table 5:** Psychopharmacological treatments use in Glycogen Storage Disease type 1 (GSD-1).

Authors, Year	Study Design	Sample (Size, Sex, Age)	Psychiatric Disorders/Symptoms	Psychiatric Medications	Symptoms Improvement (Yes/No/Not Reported)	Side Effects	Level of Evidence [25]
Dunne et al., 2019 [49]	Case report	*n* = 1 M 33 years	Psychotic symptoms	Second generation antipsychotic (olanzapine) Tricyclic antidepressant (amitriptyline)	No	Not reported	4.d

**Table 6:** Psychopharmacological treatments use in Myotonic Dystrophy type 2 (DM2).

Authors, Year	Study Design	Sample (Size, Sex, Age)	Psychiatric Disorders/Symptoms	Psychiatric Medications	Symptoms Improvement (Yes/No/Not Reported)	Side Effects	Level of Evidence [25]
Schneider et al., 2002 [50]	Case report	*n* = 1 M 26 years	Psychotic symptoms	First generation antipsychotic (flupentixol) Second generation antipsychotic (olanzapine, risperidone) Benzamide antipsychotic (amisulpride)	Not reported	Flupentixol: muscle stiffness and oculogyric crisis Olanzapine and amisulpride: raised CK, AST, ALT, GGT	4.d

**Table 7:** Psychopharmacological treatments use in Hyperkalemic Periodic Paralysis (HPP).

Authors, Year	Study Design	Sample (Size, Sex, Age)	Psychiatric Disorders/Symptoms	Psychiatric Medications	Symptoms Improvement (Yes/No/Not Reported)	Side Effects	Level of Evidence [25]
Raveendranathan et al., 2012 [51]	Case report	*n* = 1 F 26 years	Bipolar disorder	Mood stabilizer (valproate) Second generation antipsychotics (quetiapine then olanzapine) Lithium carbonate	No (valproate, quetiapine, olanzapine) Yes (lithium carbonate)	None	4.d

**Table 8:** Psychopharmacological treatments use in ^a^ RAPSN related Congenital Myasthenic Syndrome (CMS).

Authors, Year	Study Design	Sample (Size, Sex, Age)	Psychiatric Disorders/Symptoms	Psychiatric Medications	Symptoms Improvement (Yes/No/Not Reported)	Side Effects	Level of Evidence [25]
Visser et al., 2017 [52]	Case report	*n* = 1 F 42 years	Depressive symptoms	^b^ SSRI (fluoxetine)	Not reported	Worsening of episodic weakness	4.d

^a^ RAPSN: Receptor Associated Protein of the Synapse; ^b^ SSRIs: selective serotonin reuptake inhibitors.

## Data Availability

The data presented in this study are openly available in publicly accessible repositories [PubMed/MEDLINE, Embase, PsycINFO, Scopus].

## Supplementary Materials

The following are available online at https://www.mdpi.com/article/10.3390/brainsci12020176/s1, Supplementary File S1: Search strategy.

## References

[1] Deenen J.C.W., Horlings C.G.C., Verschuuren J.J.G.M., Verbeek A.L.M., van Engelen B.G.M. (2015). The Epidemiology of Neuromuscular Disorders: A Comprehensive Overview of the Literature. J. Neuromuscul. Dis..

[2] Benarroch L., Bonne G., Rivier F., Hamroun D. (2020). The 2021 version of the gene table of neuromuscular disorders (nuclear genome). Neuromuscul. Disord..

[3] Birnkrant D.J., Bushby K., Bann C.M., Apkon S.D., Blackwell A., Brumbaugh D., Case L.E., Clemens P.R., Hadjiyannakis S., Pandya S. (2018). Diagnosis and management of Duchenne muscular dystrophy, part 1: Diagnosis, and neuromuscular, rehabilitation, endocrine, and gastrointestinal and nutritional management. Lancet Neurol..

[4] Birnkrant D.J., Bushby K., Bann C.M., Alman B.A., Apkon S.D., Blackwell A., Case L.E., Cripe L., Hadjiyannakis S., Olson A.K. (2018). Diagnosis and management of Duchenne muscular dystrophy, part 2: Respiratory, cardiac, bone health, and orthopaedic management. Lancet Neurol..

[5] Birnkrant D.J., Bushby K., Bann C.M., Apkon S.D., Blackwell A., Colvin M.K., Cripe L., Herron A.R., Kennedy A., Kinnett K. (2018). Diagnosis and management of Duchenne muscular dystrophy, part 3: Primary care, emergency management, psychosocial care, and transitions of care across the lifespan. Lancet Neurol..

[6] Mercuri E., Finkel R.S., Muntoni F., Wirth B., Montes J., Main M., Mazzone E.S., Vitale M., Snyder B., Quijano-Roy S. (2018). Diagnosis and management of spinal muscular atrophy: Part 1: Recommendations for diagnosis, rehabilitation, orthopedic and nutritional care. Neuromuscul. Disord..

[7] Finkel R.S., Mercuri E., Meyer O.H., Simonds A.K., Schroth M.K., Graham R.J., Kirschner J., Iannaccone S.T., Crawford T.O., Woods S. (2018). Diagnosis and management of spinal muscular atrophy: Part 2: Pulmonary and acute care; medications, supplements and immunizations; other organ systems; and ethics. Neuromuscul. Disord..

[8] Chen M., Zhang L., Quan S. (2017). Enzyme replacement therapy for infantile-onset Pompe disease. Cochrane Database Syst. Rev..

[9] Finkel R.S., Mercuri E., Darras B.T., Connolly A.M., Kuntz N.L., Kirschner J., Chiriboga C.A., Saito K., Servais L., Tizzano E. (2017). Nusinersen versus Sham Control in Infantile-Onset Spinal Muscular Atrophy. N. Engl. J. Med..

[10] Darras B.T., Masson R., Mazurkiewicz-Bełdzińska M., Rose K., Xiong H., Zanoteli E., Baranello G., Bruno C., Vlodavets D., Wang Y. (2021). Risdiplam-Treated Infants with Type 1 Spinal Muscular Atrophy versus Historical Controls. N. Engl. J. Med..

[11] Mercuri E., Muntoni F., Baranello G., Masson R., Boespflug-Tanguy O., Bruno C., Corti S., Daron A., Deconinck N., Servais L. (2021). Onasemnogene abeparvovec gene therapy for symptomatic infantile-onset spinal muscular atrophy type 1 (STR1VE-EU): An open-label, single-arm, multicentre, phase 3 trial. Lancet Neurol..

[12] Mercuri E., Messina S., Montes J., Muntoni F., Sansone V.A., Antonaci L., Civitello M., Coratti G., de Lemus M., de Sanctis R. (2020). Patient and parent oriented tools to assess health-related quality of life, activity of daily living and caregiver burden in SMA. Rome, 13 July 2019. Neuromuscul. Disord..

[13] Messina S., Frongia A.L., Antonaci L., Pera M.C., Coratti G., Pane M., Pasternak A., Civitello M., Montes J., Mayhew A. (2019). A critical review of patient and parent caregiver oriented tools to assess health-related quality of life, activity of daily living and caregiver burden in spinal muscular atrophy. Neuromuscul. Disord..

[14] Campbell C., McColl E., McDermott M.P., Martens W.B., Guglieri M., Griggs R.C., Muscle Study Group, and TREAT-NMD (2021). Health related quality of life in young, steroid-naïve boys with Duchenne muscular dystrophy. Neuromuscul. Disord..

[15] Powell P.A., Carlton J., Rowen D., Chandler F., Guglieri M., Brazier J.E. (2021). Development of a New Quality of Life Measure for Duchenne Muscular Dystrophy Using Mixed Methods. Neurology.

[16] Davis S.E., Hynan L.S., Limbers C.A., Andersen C.M., Greene M.C., Varni J.W., Iannaccone S.T. (2010). The PedsQL^TM^ in Pediatric Patients with Duchenne Muscular Dystrophy: Feasibility, Reliability, and Validity of the Pediatric Quality of Life Inventory Neuromuscular Module and Generic Core Scales. J. Clin. Neuromuscul. Dis..

[17] Iannaccone S.T., Hynan L.S., Morton A., Buchanan R., Limbers C.A., Varni J.W. (2009). The PedsQL^TM^ in Pediatric Patients with Spinal Muscular Atrophy: Feasibility, Reliability, and Validity of the Pediatric Quality of Life Inventory^TM^ Generic Core Scales and Neuromuscular Module. Neuromuscul. Disord. NMD.

[18] Wu J.Y., Kuban K.C.K., Allred E., Shapiro F., Darras B.T. (2005). Association of Duchenne Muscular Dystrophy with Autism Spectrum Disorder. J. Child. Neurol..

[19] Ricotti V., Mandy W.P.L., Scoto M., Pane M., Deconinck N., Messina S., Mercuri E., Skuse D.D., Muntoni F.F. (2016). Neurodevelopmental, emotional, and behavioural problems in Duchenne muscular dystrophy in relation to underlying dystrophin gene mutations. Dev. Med. Child. Neurol..

[20] Thangarajh M., Spurney C.F., Gordish-Dressman H., Clemens P.R., Hoffman E.P., McDonald C.M., Henricson E.K., CINRG Investigators (2018). Neurodevelopmental Needs in Young Boys with Duchenne Muscular Dystrophy (DMD): Observations from the Cooperative International Neuromuscular Research Group (CINRG) DMD Natural History Study (DNHS). PLoS Curr..

[21] Johnson N.E., Aldana E.Z., Angeard N., Ashizawa T., Berggren K.N., Marini-Bettolo C., Duong T., Ekström A.-B., Sansone V., Tian C. (2019). Consensus-based care recommendations for congenital and childhood-onset myotonic dystrophy type 1. Neurol. Clin. Pract..

[22] Ashizawa T., Gagnon C., Groh W.J., Gutmann L., Johnson N.E., Meola G., Moxley R., Pandya S., Rogers M.T., Simpson E. (2018). Consensus-based care recommendations for adults with myotonic dystrophy type 1. Neurol. Clin. Pract..

[23] Law C., Flaherty C.V., Bandyopadhyay S. (2020). A Review of Psychiatric Comorbidity in Myasthenia Gravis. Cureus.

[24] Anglin R.E., Tarnopolsky M.A., Mazurek M.F., Rosebush P.I. (2012). The Psychiatric Presentation of Mitochondrial Disorders in Adults. J. Neuropsychiatry Clin. Neurosci..

[25] Peters M.D.J., Godfrey C., McInerney P., Munn Z., Tricco A.C., Khalil H., Aromataris E., Munn Z. (2020). Chapter 11: Scoping Reviews (2020 Version). JBI Manual for Evidence Synthesis.

[26] Tricco A.C., Lillie E., Zarin W., O’Brien K.K., Colquhoun H., Levac D., Moher D., Peters M.D.J., Horsley T., Weeks L. (2018). PRISMA Extension for Scoping Reviews (PRISMA-ScR): Checklist and Explanation. Ann. Intern. Med..

[27] Kalita J., Dongre N., Misra U.K. (2020). Myasthenic crisis due to anxiety and insomnia during COVID-19 pandemic. Sleep Med..

[28] Jordan H., Ortiz N. (2019). Management of Insomnia and Anxiety in Myasthenia Gravis. J. Neuropsychiatry Clin. Neurosci..

[29] Yamamoto A., Kimura T., Watanabe S., Yoshikawa H. (2019). Clinical characteristics of patients with myasthenia gravis accompanied by psychiatric disorders. Neurol. Clin. Neurosci..

[30] Kyllo S., McBride D., Chakos M. (2017). Safe Use of Atypical Antipsychotics in a Patient with Postpartum Psychosis and a History of Seronegative Myasthenia Gravis. Prim. Care Companion CNS Disord..

[31] She S., Yi W., Zhang B., Zheng Y. (2017). Worsening of Myasthenia Gravis After Administration of Antipsychotics for Treatment of Schizophrenia: A Case Report and Review of Literature. J. Clin. Psychopharmacol..

[32] Al-Hashel J.Y., Ismail I.I., John J.K., Ibrahim M., Ali M. (2016). Worsening of myasthenia gravis after administration of injectable long-acting risperidone for treatment of schizophrenia; first case report and a call for caution. Neuromuscul. Disord..

[33] Kim H., Hong M., Bahn G.H. (2013). Myasthenia Gravis, Schizophrenia, and Colorectal Cancer in A Patient: Long-Term Follow-Up with Medication Complexity. Psychiatry Investig..

[34] Wilson C.L., Ferguson J. (2013). Myasthenia gravis unmasked by antipsychotic medication. Prog. Neurol. Psychiatry..

[35] Chiu Y.-H., Yang A.C., Chern C.-H., How C.-K. (2011). Myasthenic Crisis May Mimic Antipsychotic-Induced Extrapyramidal Syndromes. J. Neuropsychiatry Clin. Neurosci..

[36] Alevizos B., Gatzonis S., Anagnostara C. (2006). Myasthenia Gravis Disclosed by Lithium Carbonate. J. Neuropsychiatry Clin. Neurosci..

[37] Shinkai K., Ohmori O., Ueda N., Nakamura J., Amano T., Tsuji S. (2001). A Case of Myasthenia Gravis Preceded by Major Depression. J. Neuropsychiatry Clin. Neurosci..

[38] Noda S., Murakami A., Kimura S., Minamiyama M., Katsuno M., Kuru S. (2021). Duchenne Muscular Dystrophy Successfully Treated with Aripiprazole in a Patient with Autism Spectrum Disorder Symptoms Including Irritability. Intern. Med. https://www.jstage.jst.go.jp/article/internalmedicine/advpub/0/advpub_7248-21/_article.

[39] Darmahkasih A.J., Rybalsky I., Tian C., Shellenbarger K.C., Horn P.S., Lambert J.T., Wong B.L. (2020). Neurodevelopmental, behavioral, and emotional symptoms common in Duchenne muscular dystrophy. Muscle Nerve.

[40] Lionarons J.M., Hellebrekers D.M.J., Klinkenberg S., Faber C.G., Vles J.S.H., Hendriksen J.G.M. (2019). Methylphenidate use in males with Duchenne muscular dystrophy and a comorbid attention-deficit hyperactivity disorder. Eur. J. Paediatr. Neurol..

[41] Lee A.J., Buckingham E.T., Kauer A.J., Mathews K.D. (2018). Descriptive phenotype of obsessive compulsive symptoms in males with Duchenne Muscular Dystrophy. J. Child. Neurol..

[42] Hendriksen J.G.M., Klinkenberg S., Collin P., Wong B., Niks E.H., Vles J.S. (2016). Diagnosis and treatment of obsessive compulsive behavior in a boy with Duchenne muscular dystrophy and autism spectrum disorder: A case report. Neuromuscul. Disord..

[43] Lambert J.T., Darmahkasih A.J., Horn P.S., Rybalsky I., Shellenbarger K.C., Tian C., Wong B.L. (2020). Neurodevelopmental, behavioral, and emotional symptoms in Becker muscular dystrophy. Muscle Nerve.

[44] Santos C.F. (2019). First psychotic episode in an adult with Becker muscular dystrophy. Rev. Bras. Psiquiatr..

[45] Chaichana K.L., Buffington A.L.H., Brandes M., Edwin D., Lee H.B. (2007). Treatment of Psychiatric Comorbidities in a Patient with Becker Muscular Dystrophy. ProQuest. Psychosomatics.

[46] Cozart B., Vera J.D., Denham J.D., Whiting W.L. (2018). Treatment of Depression with Duloxetine in Mitochondrial Encephalopathy, Lactic Acidosis, and Stroke-Like Episodes. Clin. Neuropharmacol..

[47] Grover S., Padhy S.K., Das C.P., Vasishta R.K., Sharan P., Chakrabarti S. (2006). Mania as a first presentation in mitochondrial myopathy. Psychiatry Clin. Neurosci..

[48] Lacey C.J., Salzberg M.R. (2008). Obsessive-compulsive disorder with mitochondrial disease. Psychosomatics.

[49] Dunne T.F., Geberhiwot T., Jones R. (2019). Acute psychosis in glycogen storage disease: A rare but severe complication. BMJ Case Rep..

[50] Schneider C., Pedrosa Gil F., Schneider M., Anetseder M., Kress W., Müller C.R. (2002). Intolerance to neuroleptics and susceptibility for malignant hyperthermia in a patient with proximal myotonic myopathy (PROMM) and schizophrenia. Neuromuscul. Disord..

[51] Raveendranathan D., Babu G.N., Desai G., Chandra P.S. (2012). Bipolar Disorder Co-Occurring with Periodic Paralysis: A Case Report. J. Neuropsychiatry Clin. Neurosci..

[52] Visser A.C., Laughlin R.S., Litchy W.J., Benarroch E.E., Milone M. (2017). Rapsyn congenital myasthenic syndrome worsened by fluoxetine. Muscle Nerve.

[53] Kulaksizoglu I.B. (2007). Mood and Anxiety Disorders in Patients with Myasthenia Gravis: Aetiology, Diagnosis and Treatment. CNS Drugs.

[54] Van der Velden B.G., Okkersen K., Kessels R.P., Groenewoud J., van Engelen B., Knoop H., Raaphorst J. (2019). Affective symptoms and apathy in myotonic dystrophy type 1 a systematic review and meta-analysis. J. Affect. Disord..

[55] Anglin R.E., Garside S.L., Tarnopolsky M.A., Mazurek M.F., Rosebush P.I. (2012). The psychiatric manifestations of mitochondrial disorders: A case and review of the literature. J. Clin. Psychiatry.

[56] Rahbek J., Steffensen B.F., Bushby K., de Groot I.J.M. (2015). Care for a Novel Group of Patients—Adults with Duchenne Muscular Dystrophy. In Proceedings of the 206th ENMC International Workshop, Naarden, The Netherlands, 23–25 May 2014. Neuromuscul. Disord..

[57] Quinlivan R., Messer B., Murphy P., Astin R., Mukherjee R., Khan J., Emmanuel A., Wong S., Kulshresha R., Willis T. (2021). Adult North Star Network (ANSN): Consensus Guideline for the Standard of Care of Adults with Duchenne Muscular Dystrophy. J. Neuromuscul. Dis..

[58] De Vries M.C., Brown D.A., Allen M.E., Bindoff L., Gorman G.S., Karaa A., Keshavan N., Lamperti C., McFarland R., Ng Y.S. (2020). Safety of drug use in patients with a primary mitochondrial disease: An international Delphi-based consensus. J. Inherit. Metab. Dis..

