# Supplementary material for: Psychopharmacological Treatments for Mental Disorders in Patients with Neuromuscular Diseases: A Scoping Review
Source: Brain Sci. 2022 Jan 28;12(2):176. doi: 10.3390/brainsci12020176 (PMC8870619; doi:10.3390/brainsci12020176)
Supplement: Supplementary file 1 [file brainsci-12-00176-s001.zip › brainsci-1513467-supplementary.pdf]

File S1. Search strategy for PubMed/MEDLINE

Search string

((((((((((((((((((((((Psychotropic Drugs[MeSH Terms]) OR (Psychopharmacology[MeSH Terms])) OR (Anti-Anxiety Agents[MeSH Terms])) OR (Antidepressive Agents[MeSH Terms])) OR (Antimanic Agents[MeSH Terms])) OR (Antipsychotic Agents[MeSH Terms])) OR (Central Nervous System Stimulants[MeSH Terms])) OR (Mental Disorders[MeSH Terms])) OR (Anxiety Disorders[MeSH Terms])) OR (Obsessive-Compulsive Disorder[MeSH Terms])) OR (Panic Disorder[MeSH Terms])) OR (Bipolar Disorder[MeSH Terms])) OR (Mood Disorders[MeSH Terms])) OR (Depressive Disorder[MeSH Terms])) OR (Neurodevelopmental Disorders[MeSH Terms])) OR (Attention Deficit Disorder with Hyperactivity[MeSH Terms])) OR (Conduct Disorder[MeSH Terms])) OR (Autism Spectrum Disorder[MeSH Terms])) OR (Psychotic Disorders[MeSH Terms])) OR (Schizophrenia[MeSH Terms])) AND ((((((((((((((((((((((Neuromuscular Diseases[MeSH Terms]) OR (Muscular Atrophy, Spinal[MeSH Terms])) OR (Muscular Diseases[MeSH Terms])) OR (Mitochondrial Myopathies[MeSH Terms])) OR (Muscular Disorders, Atrophic[MeSH Terms])) OR (Muscular Dystrophies[MeSH Terms])) OR (Distal Myopathies[MeSH Terms])) OR (Muscular Dystrophies, Limb-Girdle[MeSH Terms])) OR (Muscular Dystrophy, Duchenne[MeSH Terms])) OR (Muscular Dystrophy, Emery-Dreifuss[MeSH Terms])) OR (Muscular Dystrophy, Facioscapulohumeral[MeSH Terms])) OR (Muscular Dystrophy, Oculopharyngeal[MeSH Terms])) OR (Myopathies, Structural, Congenital[MeSH Terms])) OR (Myositis[MeSH Terms])) OR (Myotonic Disorders[MeSH Terms])) OR (Myotonia Congenita[MeSH Terms])) OR (Myotonic Dystrophy[MeSH Terms])) OR (Paralyses, Familial Periodic[MeSH Terms])) OR (Neuromuscular Junction Diseases[MeSH Terms])) OR (Myasthenia Gravis[MeSH Terms])) OR (Myasthenic Syndromes, Congenital[MeSH Terms])) OR (Glycogen Storage Disease[MeSH Terms]))) NOT ((fibromyalgia[MeSH Terms]) OR (Peripheral Nervous System Diseases[MeSH Terms]))

Search terms

Psychotropic Drugs, Psychopharmacology, Anti-Anxiety Agents, Antidepressive Agents, Antimanic Agents, Antipsychotic Agents, Central Nervous System Stimulants, Mental Disorders, Anxiety Disorders, Obsessive-Compulsive Disorder, Panic Disorder, Bipolar Disorder, Mood Disorders, Depressive Disorder Neurodevelopmental Disorders, Attention Deficit Disorder with Hyperactivity, Conduct Disorder, Autism Spectrum Disorder, Psychotic Disorders, Schizophrenia, Neuromuscular Diseases, Muscular Atrophy, Spinal Muscular Diseases, Mitochondrial Myopathies, Muscular Disorders Atrophic, Muscular Dystrophies, Distal Myopathies, Muscular Dystrophies Limb-Girdle, Muscular Dystrophy Duchenne, Muscular Dystrophy Emery-Dreifuss, Muscular Dystrophy Facioscapulohumeral, Muscular Dystrophy Oculopharyngeal, Myopathies Structural Congenital, Myositis, Myotonic Disorders, Myotonia Congenita, Myotonic Dystrophy, Paralyses Familial Periodic, Neuromuscular Junction Diseases, Myasthenia Gravis, Myasthenic Syndromes Congenital, Glycogen Storage Disease
